# Antifungal Activity and Possible Mechanism of Action of the Zn (II) Complex With a Schiff Base Against Fluconazole‐Resistant Strains of *Candida* spp. and *Cryptococcus neoformans*

**DOI:** 10.1002/cbdv.71538

**Published:** 2026-07-26

**Authors:** Lívia Gurgel do Amaral Valente Sá, Cecília Rocha da Silva, João Batista de Andrade Neto, Daniel Sampaio Rodrigues, Lara Elloyse Almeida Moreira Gomes, Vitória Pessoa de Farias Cabral, Wildson Max Barbosa da Silva, Solange de Oliveira Pinheiro, Jordana Lima Braga, Bruno Coêlho Cavalcanti, Manoel Odorico de Moraes, Thais Lima Ferreira, Elaine Aires de Lima, Ana Carolina Medeiros de Oliveira, Hélio Vitoriano Nobre Júnior

**Affiliations:** ^1^ Department of Clinical and Toxicological Analysis, Faculty of Pharmacy, Laboratory of Bioprospection in Antimicrobial Molecules (LABIMAN) Federal University of Ceará (UFC) Fortaleza Ceará Brazil; ^2^ Laboratory For Bioprospecting in Antimicrobial Molecules (LABIMAN), Center For Drug Research and Development (NPDM) Federal University of Ceará (UFC) Fortaleza Ceará Brazil; ^3^ Christus University (Unichristus) Fortaleza Ceará Brazil; ^4^ Chemistry Course Vale Do Acaraú State University (UVA) Sobral Ceará Brazil; ^5^ Graduate Program in Biotechnology (PPGB) Federal University of Ceará (UFC) Sobral Ceará Brazil; ^6^ Center For Sciences and Technology Chemistry Course Ceará State University (UECE) Fortaleza Ceará Brazil; ^7^ Drug Research and Development Center (NPDM), Postgraduate Program in Translation Medicine Federal University of Ceará (UFC) Fortaleza Ceará Brazil; ^8^ Department of Physiology and Pharmacology Federal University of Ceará (UFC) Fortaleza Ceará Brazil

**Keywords:** *Candida* spp, *Cryptococcus neoformans*, mechanism of action, Schiff base, zinc

## Abstract

The objective of this study was to investigate the antifungal activity of the vanillin‐derived Schiff base complexed with zinc (II) (SBZ) against fluconazole‐resistant strains of *Candida* spp. and *Cryptococcus neoformans*. The broth microdilution assay was performed to determine the minimum inhibitory concentration (MIC). The checkerboard assay was used to evaluate the type of pharmacological interaction of the combination of SBZ with azoles. Flow cytometry and alkaline comet assays were performed to investigate the possible action mechanism of SBZ against *Candida albicans* and *Cryptococcus neoformans*. The MIC values of SBZ for *Candida* spp. ranged from 24 to 74.7 µg/mL and for *C. neoformans* from 32 to 53.3 µg/mL. The combination of SBZ with both fluconazole and itraconazole resulted in additive and indifferent interactions, respectively. The mechanism of action appears to involve mitochondrial depolarization, increased oxidative stress, and DNA damage, leading to apoptosis. Thus, the SBZ complex shows promising in vitro antifungal activity, and its combination with azoles may contribute to optimizing the efficacy of these drugs, justifying future in vivo investigations.

## Graphical abstract

Addressing the global challenge of antifungal resistance, this work presents a vanillin‐derived Schiff base complexed with zinc (II) (SBZ) with potent activity against fluconazole‐resistant *Candida* spp. and *Cryptococcus neoformans*. By activating an apoptotic pathway, ranging from mitochondrial depolarization and oxidative stress to fungal DNA fragmentation, SBZ can overcome antifungal resistance. Furthermore, its promising additive effects with traditional azoles offer a multi‐targeted strategy for more effective combination therapies against priority fungal pathogens.

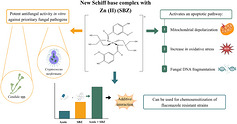

## 1 Introduction

Human infections caused by fungi, although receiving less attention than bacterial and viral infections, have significant morbidity and mortality rates, causing up to 2 million deaths per year worldwide and affecting approximately 150 million people [1, 2]. Globally, some invasive fungal infections stand out for their alarming numbers, such as invasive candidiasis, with approximately 700 000 cases per year, and cryptococcal meningitis, with approximately 250 000 yearly cases [1, 2].

In addition to their widespread prevalence, these infections can also be difficult to treat, mostly due to the weakened immunity of the affected individuals and also due to the increase in the number of strains resistant to antifungal drugs [1]. In the case of *Candida* spp. species, resistance to azoles, particularly fluconazole (FLC), is one of the main limitations in the treatment of candidiasis. In addition, the emerging and multidrug‐resistant species *Candida auris* is also cause for concern [3].

In view of the World Health Organization's list of priority fungal pathogens [4], it is important for new studies to seek therapeutic alternatives against *Candida* species, including *C. auris*, and also against *Cryptococcus neoformans*. As a therapeutic alternative, the properties of metal ion‐based antimicrobials have been investigated [5]. Schiff bases (SB) are studied because they have different applications and biological properties [6]. It is also reported that Schiff bases complexed with metal ions have better biological activity compared to the use of isolated metal ions [7, 8].

Schiff bases complexed with zinc (II) ions may be a promising alternative, since they have different pharmaceutical properties, including antimicrobial activity against Gram‐positive and Gram‐negative bacteria, as well as *Candida albicans* yeast [8, 9, 10]. Recent literature has demonstrated that newly synthesized Zn(II) complexes possess potent inhibitory activity against fungal pathogens, such as *Candida albicans* and *Aspergillus niger*, owing to their interaction with microbial cell membranes and DNA‐binding capacity [11, 12].

In this sense, metal complexes derived from Schiff bases, especially those containing Zn(II), have emerged as promising candidates in the development of antifungal agents due to their combination of favorable structural and functional properties [13]. Schiff bases act as versatile ligands, capable of stabilizing the metal ion and modulating its lipophilicity, which can facilitate permeation through the fungal cell membrane [14]. Zn(II), in turn, exhibits relatively low toxicity in biological systems and high affinity for nitrogen‐ and oxygen‐containing sites, allowing interactions with essential biomolecules such as proteins and nucleic acids [15, 16].

Furthermore, zinc coordination can promote changes in the indirect redox reactivity of the system, contributing to the generation of intracellular oxidative stress [17]. Together, these characteristics can lead to the disruption of critical cellular processes, such as mitochondrial function, membrane integrity, and redox homeostasis [17, 18], resulting in the inhibition of growth or death of fungal cells. Thus, Zn(II) complexes with Schiff bases represent a rational and promising strategy in the search for new antifungals with differentiated mechanisms of action.

**FIGURE 1:**
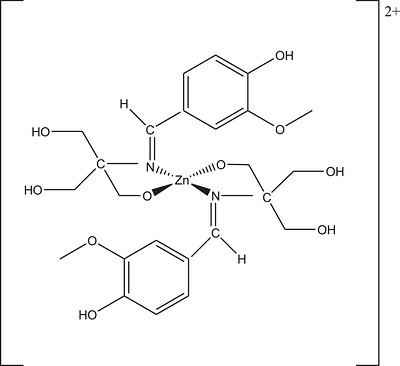
Representative structure of the complex SBZ.

The antifungal activity of the Zn (II) complex with the Schiff base derived from vanillin was already reported by our group in a previous study against *Candida albicans, Candida auris*, and *Cryptococcus neoformans* [19]. Continuing this work, in the present study we investigated the activity of the Zn (II) complex with the vanillin‐derived Schiff base (SBZ) (Figure 1) against a larger number of *Candida* spp. strains resistant to FLC, and *Cryptococcus neoformans*, as well as to determine the type of interaction that the SBZ complex presents when combined with azoles (FLC and itraconazole [ITR]) against these yeasts. Finally, we also investigated the possible mechanism of action of the SBZ complex against *Candida albicans* and *Cryptococcus neoformans*.

The study of imines, especially Schiff bases, coordinated to zinc ions is relevant due to the ability of these complexes to interact selectively with biological systems. Zinc, being an essential metal with low toxicity, favors safe pharmacological applications. Coordination with imines can modify electronic and structural properties, enhancing antifungal activity. Thus, these complexes become promising candidates for the development of new therapeutic agents.

Additionally, the novelty of this study lies in the description and antifungal evaluation of the SBZ complex, a previously unreported Zn(II)‐coordinated Schiff base derivative. In this way, the originality of this work stems from its integrated approach, combining standard antifungal assays with detailed mechanistic analyses. By evaluating these cellular effects in *Cryptococcus neoformans* and in *Candida albicans* and non*‐albicans* species, this research seeks to provide a comprehensive understanding of the biological impact of Zn(II) complexes, filling a gap in the development of bioactive metal‐based therapies.

## 2 Results and Discussion

### 2.1 In Vitro Antifungal Activity of the SBZ Complex

With regard to the Schiff base (SB), all *Candida* spp. strains had MIC > 1024 µg/mL, while *Cryptococcus neoformans* strains had MIC between 853.3 and 512 µg/mL. With regard to the SBZ complex, *Candida* spp. strains presented MIC between 80 and 24 µg/mL, while *Cryptococcus neoformans* strains presented MIC between 53.3 and 32 µg/mL, as shown in Table 1.

**TABLE 1:** Antifungal activity of Schiff base (SB) and SBZ complex against *Candida* spp. and *Cryptococcus neoformans* strains.

	MIC 50%			
Strains	SB (µg/mL)	SBZ (µg/mL)	FLC (µg/mL)	ITRA (µg/mL)
*Candida parapsilosis* ATCC 22019	> 1024	64	1	0.5
*Candida krusei* ATCC 6258	> 1024	37.3	26.7	0.5
*Candida auris* 01256P CDC	> 1024	64	1.5	0.5
*Candida albicans* 1*	> 1024	74.7	16	1.7
*Candida albicans* 2	> 1024	26.7	64	4
*Candida tropicalis*	> 1024	32	26.7	0.9
*Candida parapsilosis*	> 1024	80	8	0.2
*Candida glabrata*	> 1024	24	64	4
*Cryptococcus neoformans* 1*	512	32	0,5	0.3
*Cryptococcus neoformans* 2	512.0	32	4	0.8
*Cryptococcus neoformans* 3	682.7	32	4	0.8
*Cryptococcus neoformans* 4	682.7	53.3	2	0.4
*Cryptococcus neoformans* 5	853.3	42.7	2	0.3
*Cryptococcus neoformans* 6	768.0	53.3	2	0.3
*Cryptococcus neoformans* 7	682.7	32	2	0.4

Minimum inhibitory concentration (MIC) values capable of inhibiting 50% of the growth of *Candida* spp. and *Cryptococcus neoformans* strains, after incubation for 24 and 72 h, respectively, with Schiff base (SB), Zn (II) complex with Schiff base (SBZ), fluconazole (FLC), and itraconazole (ITRA) in µg/mL. MIC values were obtained by the arithmetic mean of the triplicate results. * Representative strain used in the flow cytometry and alkaline comet assay.

In our study, we observed that the antifungal activity of SBZ was better than that of SB. Other studies have also found this characteristic when coordinating transition metal ions, such as Zn (II), in a Schiff base [20, 21, 22, 23]. Previous studies have shown that Zn (II) complexes with Schiff bases exhibit antifungal activity against different *Candida* species, with MICs ranging from 8 to 256 µg/mL [21, 23, 24, 25]. However, these studies investigated the antifungal potential of SBZ in strains sensitive to FLC.

Our results demonstrate that even in the presence of FLC‐resistant *Candida* spp. strains, SBZ exhibits antifungal activity. Our findings corroborate those of Geweely [26] who synthesized metal (II) complexes with a Schiff base derived from cinnamaldehyde and evaluated their biological activity against six pathogenic *Candida* species, finding MICs ranging from 1.25 to 12.5 µg/mL against different FLC‐resistant *Candida* species. The Zn (II) complex was the most effective, showing a strong inhibitory effect. In the studies by Dar et al. [27] and Dar et al. [28], MIC values ranging from 31.25 to 250 µg/mL were found against FLC‐resistant strains of *Candida albicans*. The difference in MIC values observed in the studies by Dar et al. [27] and Dar et al. [28] can be attributed to differences in the structures of the inorganic complexes investigated in each study.

Small variations in the ligand (Schiff base), central metal ion, complex geometry, degree of hydration, and chemical stability can alter lipophilicity, cell penetration, and interaction with fungal targets, resulting in different MICs [14].

Regarding the antifungal activity of SBZ in *Cryptococcus neoformans*, our results corroborate those of Oliveira Pinheiro et al. [19], which shows that the complexation of zinc (II) ions in the VTRIS Schiff base improved antifungal activity against this pathogen, which is associated with severe cases of lung infections and meningitis in immunocompromised individuals [29]. Due to the limited data available in the literature, further studies should be conducted to evaluate the efficacy of SBZ in anti‐cryptococcal therapy.

The safety profile of the compounds was preliminarily assessed using the Artemia salina lethality bioassay in a work published by our group in 2025 [19]. The Zn(II) complex and its corresponding Schiff base ligand exhibited low toxicity, with LC50 values of 543.88 µg/mL and 698.56 µg/mL, respectively [19]. These results indicate that the concentrations required to exert antifungal effects are significantly lower than the threshold for acute toxicity in this model, supporting the potential of these complexes for further drug development.

### 2.2 Interaction Between the SBZ Complex and Antifungal Agents

In the combination of SBZ with FLC, there were 80% additive interactions (*n* = 12) and 20% indifferent interactions (*n* = 3), as shown in Table 2. There were no synergistic or antagonistic interactions.

**TABLE 2:** Determination of FICI and the effect of combining the complex with fluconazole against strains of *Candida* spp. and *Cryptococcus neoformans*.

	MIC^a^ (µg/mL) isolated		MIC^b^ (µg/mL) combined			
Strains	SBZ	FLC	SBZ	FLC	FICI^c^	Interpretation
*Candida parapsilosis* ATCC 22019	64	1	32	0.5	1.0	Additive
*Candida krusei* ATCC 6258	37.3	26.7	24.9	17.8	1.3	Indifferent
*Candida auris* 01256P CDC	64	1.5	32	0.7	1.0	Additive
*Candida albicans* 1	74.7	16	37.4	8	1.0	Additive
*Candida albicans* 2	26.7	64	8.9	21.3	0.7	Additive
*Candida tropicalis*	32	26.7	16	13.3	1.0	Additive
*Candida parapsilosis*	80	8	33.3	3.3	0.8	Additive
*Candida glabrata*	24	64	12	32	1.0	Additive
*Cryptococcus neoformans* 1	32	0.5	16	0.3	1.0	Additive
*Cryptococcus neoformans* 2	32	4	16	2	1.0	Additive
*Cryptococcus neoformans* 3	32	4	21.3	2.7	1.4	Indifferent
*Cryptococcus neoformans* 4	53.3	2	22.2	0.8	0.8	Additive
*Cryptococcus neoformans* 5	42.7	2	21.3	1	1.0	Additive
*Cryptococcus neoformans* 6	53.3	2	26.7	1	1.0	Additive
*Cryptococcus neoformans* 7	32	2	21.3	1.3	1.4	Indifferent

^a^ Minimum inhibitory concentration (MIC) of 50% for the SBZ complex and fluconazole (FLC) alone.

^b^ Minimum inhibitory concentration (MIC) of SBZ complex and fluconazole (FLC) after combination.

^c^ Fractional inhibitory concentration index (FICI). MIC values were obtained by the arithmetic mean of the triplicate results.

In the combination of SBZ with ITRA, there were 53.3% additive interactions (*n* = 8) and 46.7% indifferent interactions (*n* = 7), as shown in Table 3. There were no synergistic or antagonistic interactions.

**TABLE 3:** Determination of FICI and the effect of combining the complex with itraconazole against strains of *Candida* spp. and *Cryptococcus neoformans*.

	MIC^a^ (µg/mL) isolated		MIC^b^ (µg/mL) combined			
Strains	SBZ	ITRA	SBZ	ITRA	FICI^c^	Interpretation
*Candida parapsilosis* ATCC 22019	64	0.5	21.3	0.2	0.7	Additive
*Candida krusei* ATCC 6258	37.3	0.5	15.5	0.2	0.8	Additive
*Candida auris* 01256P CDC	64	0.5	21.3	0.2	0.7	Additive
*Candida albicans* 1	74.7	1.7	31.1	0.7	0.8	Additive
*Candida albicans* 2	26.7	4	11.2	1.7	0.8	Additive
*Candida tropicalis*	32	0.9	16	0.5	1.0	Additive
*Candida parapsilosis*	80	0.2	20	0.5	2.75	Indifferent
*Candida glabrata*	24	4	10	1.7	0.8	Additive
*Cryptococcus neoformans* 1	32	0.3	16	0.2	1.2	Indifferent
*Cryptococcus neoformans* 2	32	0.8	21.3	0.5	1.3	Indifferent
*Cryptococcus. neoformans* 3	32	0.8	21.3	0.5	1.3	Indifferent
*Cryptococcus. neoformans* 4	53.3	0.4	26.7	0.2	1.0	Additive
*Cryptococcus. neoformans* 5	42.7	0.3	21.3	0.2	1.2	Indifferent
*Cryptococcus neoformans* 6	53.3	0.3	26.7	0.2	1.2	Indifferent
*Cryptococcus neoformans* 7	32	0.4	32	0.4	2.0	Indifferent

^a^ Minimum inhibitory concentration (MIC) of SBZ complex and itraconazole (ITRA) alone.

^b^ Minimum inhibitory concentration (MIC) of SBZ complex and itraconazole (ITRA) after combination.

^c^ Fractional inhibitory concentration index (FICI). MIC values were obtained by the arithmetic mean of the triplicate results.

Regarding the interaction of SBZ with FLC, our results showed a predominance of additive interactions and few indifferent interactions against FLC‐resistant *Candida* spp. strains. Dar et al. [28] evaluated the interaction of a Schiff base complex with zinc (II) against *Candida albicans* strains sensitive (*n* = 8) and resistant (*n* = 3) to FLC, observing a predominance of synergistic interactions, followed by additive interactions and only one indifferent interaction. All resistant strains showed synergistic interactions.

The difference obtained between the two compounds can be explained, in part, by the fact that we used a larger number of strains, increasing the biological variability analyzed. In addition, the distinct composition of the inorganic complex in relation to the reference study may have influenced its properties and consequently the experimental results. However, additive interactions are also relevant, since in this case there was a certain decrease in the MIC of FLC, indicating that SBZ can be used for chemosensitization of FLC‐resistant strains, with further studies needed to confirm this. In addition, there were no antagonistic interactions, showing that it is safe to combine SBZ with FLC.

There are no other studies in the literature on the interaction of SBZ with FLC against *Cryptococcus neoformans* and SBZ with ITRA against *Candida* spp. and *Cryptococcus neoformans*, which highlights the importance of the results found here. Our results show there is an advantage in combining SBZ with ITRA, since it predominantly presents additive interactions, and that it is safe, as it does not present antagonistic interactions. This serves as a basis for future studies to further investigate the interaction between these drugs.

### 2.3 Possible Mechanism of Action

#### 2.3.1 Cell Viability

**FIGURE 2:**
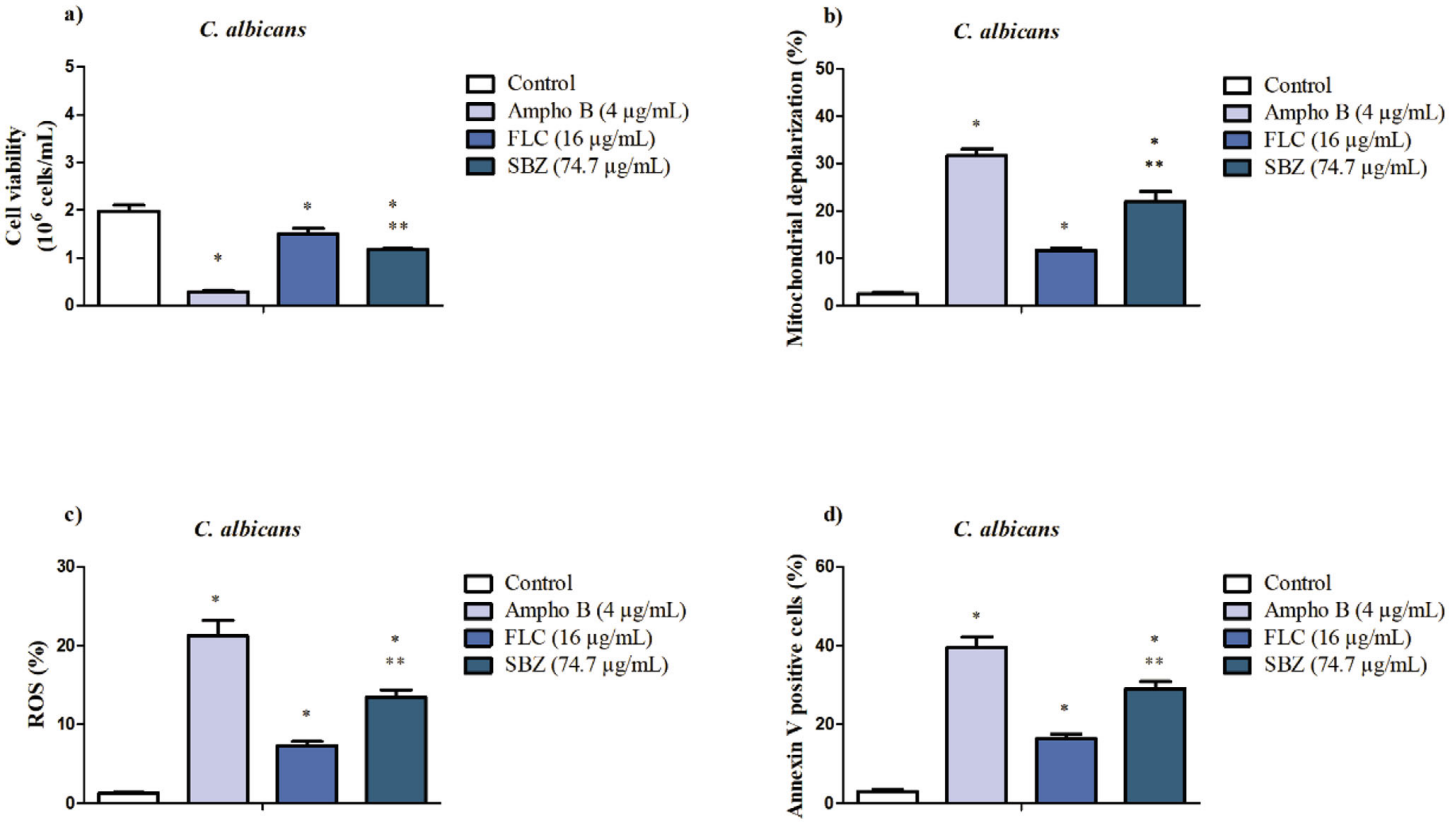
Possible antifungal mechanism of action of SBZ against *Candida albicans* by flow cytometry. After treating *Candida albicans* (*n* = 1) and with the MIC of SBZ and fluconazole (FLC) for 24 h, cell viability (a), mitochondrial depolarization (b), ROS production (c) and Anexin V‐labeled cells (d) of each species were evaluated, respectively. Amphotericin B (Ampho B) was used as death control and the control was untreated fungal cells. *p < 0.05 compared to the control, submitted to ANOVA followed by the Newman–Keuls test.

Treatment with SBZ and FLC significantly reduced (*p* < 0.05) the cell viability of *Candida albicans* to 1. 17 × 10^6^ ± 0.02 cells/mL and 1.50 × 10^6^ ± 0.11 cells/mL, respectively, compared to the control (1.96 × 10^6^ ± 0.12 cells/mL). Treatment with SBZ was more significant than treatment with FLC against this fluconazole‐resistant strain of *Candida albicans*. The death control, amphotericin B (Ampho B), reduced cell viability to 0.29 × 10^6^ ± 0.02 cells/mL (Figure 2a).

**FIGURE 3:**
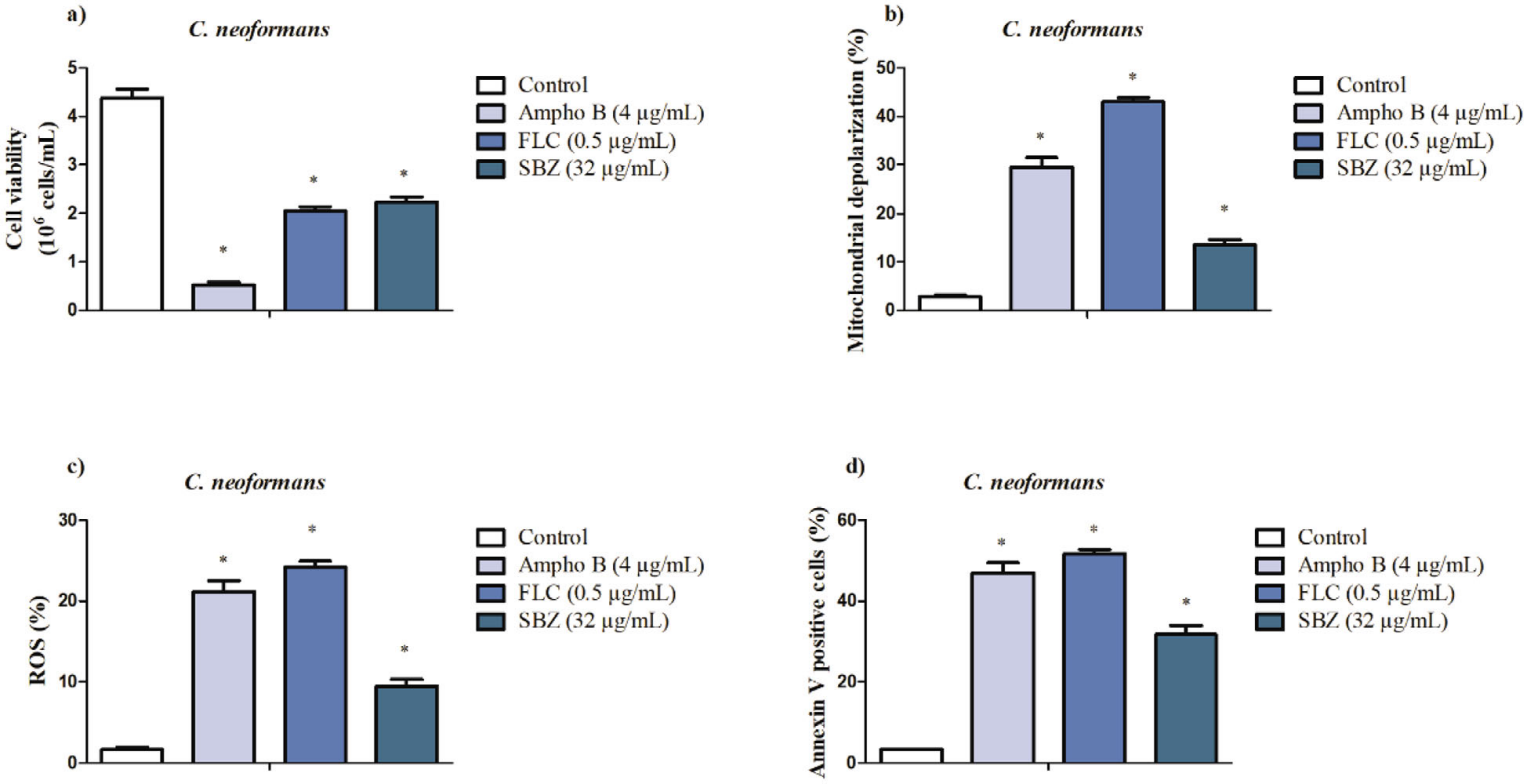
Possible antifungal mechanism of action of SBZ against *Cryptococcus neoformans* by flow cytometry. After treating *Cryptococcus neoformans* (*n* = 1) with the MIC of SBZ and fluconazole (FLC) for 24 h, cell viability (a), mitochondrial depolarization (b), ROS production (c) and Anexin V‐labeled cells (d) of each species were evaluated, respectively. Amphotericin B (Ampho B) was used as death control in relation to untreated fungal cells. *p < 0.05 compared to the control, submitted to ANOVA followed by the Newman–Keuls test.

As for *Cryptococcus neoformans*, SBZ and FLC significantly reduced (*p* < 0.05) cell viability to 2.24 × 10^6^ ± 0.10 cells/mL and 2.05 × 10^6^ ± 0.10 cells/mL, respectively, compared to the control (4.38 × 10^6^ ± 0.19 cells/mL). Ampho B reduced cell viability to 0.29 × 10^6^ ± 0.02 cells/mL (Figure 3a).

#### 2.3.2 Mitochondrial Depolarization

Treatment with SBZ and FLC significantly altered (*p* < 0.05) the mitochondrial transmembrane potential of *Candida albicans*, increasing mitochondrial depolarization by 22% and 11.7%, respectively, compared to the control. Treatment with SBZ was more significant than treatment with FLC. Ampho B increased mitochondrial depolarization by 31.7% (Figure 2b).

The increase in mitochondrial depolarization of *Cryptococcus neoformans* was also significant after treatment with SBZ (13.6%) and FLC (43%) compared to the control. Ampho B increased mitochondrial depolarization by 29.6% (Figure 3b).

#### 2.3.3 ROS Production

There were significant increases (*p* < 0.05) in ROS production in *Candida albicans* after treatment with SBZ (13.5%) and FLC (7.3%), compared to the control. Treatment with SBZ was more significant than treatment with FLC. Treatment with Ampho B increased ROS production by 21.3% (Figure 2c).

With *Cryptococcus neoformans*, there were also significant increases in ROS production in the treatments with SBZ (9.5%) and FLC (24.2%), compared to the control. Ampho B increased ROS production by 21.2% (Figure 3c).

#### 2.3.4 Cells Stained With Annexin V

Cells positively stained with Annexin V are indicative of apoptosis. Thus, there were significant increases (*p* < 0.05) in *Candida albicans* cells stained with Annexin V after treatment with SBZ (29.1%) and FLC (16.4%) compared to the control. Treatment with SBZ was more significant than treatment with FLC. Treatment with Ampho B resulted in a 39.6% increase (Figure 2d).

There were also significant increases in *Cryptococcus neoformans* cells labeled with Annexin V after treatment with SBZ (31.7%) and FLC (51.7%), compared to the control. Treatment with Ampho B resulted in a 47% increase (Figure 3d).

#### 2.3.5 Damage to Yeast DNA

**FIGURE 4:**
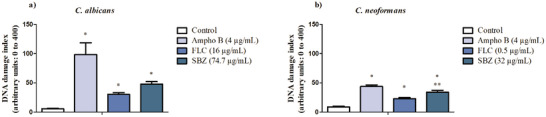
Alkaline comet assay of *Candida albicans* (a) and *Cryptococcus neoformans* (b) cells exposed to SBZ. DNA damage caused by the treatments with the respective MIC values of SBZ and fluconazole (FLC) of *Candida albicans* (*n* = 1) and *Cryptococcus neoformans* (*n* = 1), control (untreated cells) and Amphotericin B (Ampho B), as death control, after exposure for 24 h. *p < 0.05 compared to control, submitted to ANOVA followed by the Newman–Keuls test.

Treatment with SBZ and FLC significantly increased (*p* < 0.05) DNA damage in *Candida albicans*, with damage indices of 47.5 and 30, respectively, compared to the control. Treatment with Ampho B resulted in a damage index of 98.3. In *Cryptococcus neoformans*, the treatments also significantly increased DNA damage in the yeast, with damage indices of 33.8 and 22.7, respectively, compared to the control, with SBZ treatment being more significant than FLC treatment. Treatment with Ampho B resulted in a damage index of 43.7 (Figure 4).

Regarding the mechanism of action of SBZ against *Candida albicans* and *Cryptococcus neoformans* yeasts, a decrease in cell viability was observed due to an increase in mitochondrial depolarization, increased ROS production, and increased microbial DNA damage, leading to death by apoptosis. It is worth noting that in the case of *Candida albicans*, we observed that treatment with SBZ caused more significant damage than with FLC. Given that this strain is resistant to FLC, treatment with SBZ proved promising against this pathogen.

As summarized in Table 4, the observed depolarization of the mitochondrial membrane potential identifies this organelle as a primary target, a process facilitated by the increased lipophilicity of the Zn(II) complex upon coordination. This dysfunction triggers a pro‐oxidant state characterized by the overproduction of intracellular ROS. The subsequent oxidative stress leads to systemic damage, where the observed DNA fragmentation and Annexin V positivity serve as hallmarks of apoptosis. Therefore, the SBZ complex exerts its effect through a cascade of events, from membrane permeation and mitochondrial disruption to oxidative‐driven programmed cell death.

**TABLE 4:** Summary of the core findings for the SBZ complex.

Assay	Core findings
In vitro antifungal activity (MIC)	SBZ showed higher potency against *Candida* spp. and *Cryptococcus neoformans* (MIC 24–80 µg/mL) compared to the ligand (SB > 512 µg/mL), including efficacy against fluconazole‐resistant strains.
Drug interaction	Predominantly additive interactions. The absence of antagonism suggests potential for combination therapy and chemosensitization.
Cell viability	SBZ significantly reduced cell viability and increased Annexin V staining (∼29%–31%), identifying apoptosis as the main cell death pathway.
Mitochondrial and oxidative stress	Induction of mitochondrial membrane depolarization and ROS overproduction, indicating mitochondrial collapse as a key early event.
Damage to yeast DNA (Comet assay)	High DNA damage index and DNA fragmentation, confirming systemic molecular damage.

The incorporation of transition metals into ligands, such as Schiff bases, promotes lipophilicity to the metal center, due to the chelation effect, allowing its entry into the fungal cell membrane [20, 22, 30], in addition to the fact that zinc (II) ions are trace elements necessary for the metabolic function of cells, including fungi, which also favors their entry [31].

In addition, it is reported that zinc (II) ion complexes can interact with proteins and DNA, causing damage as well as increasing ROS production and hence oxidative stress in fungal cells, interfering with cellular respiration, contributing to further molecular damage and loss of membrane integrity, leading to cell death [24, 25, 31], as also observed in our study. Schiff base complexes with zinc (II) also have the ability to inhibit the growth of microorganisms by cleaving their DNA [32].

Geweely [26] investigated the activity of different Schiff base complexes against *Candida albicans, Candida parapsilosis, Candida tropicalis*, and *Candida glabrata*. The complex with the most promising activity was that with zinc (II) ions, which decreased the percentage of viable fungal cells and cellular respiration, in addition to interfering with the sugar content present in the cell membrane of *Candida* spp. and modulating proteins in its cell wall the latter being the main target of the zinc (II) metal complex according to the authors.

Dar et al. [27] demonstrated that a Schiff base complex obtained by the condensation of 2‐hydroxynaphthaldehyde and tryptamine, and 1,10‐phenanthroline (1,10‐phen) as a secondary ligand with zinc (II) as the metal center, was able to inhibit the proton pump of the cell membrane of FLC‐sensitive and FLC‐resistant strains of *Candida albicans*. Additionally, an in silico study revealed that a Schiff base complex with zinc (II) exhibited low binding energy and excellent interaction affinity with the sterol 14α‐demethylase protein of *Candida albicans* [33]. Considering the observed activity of this complex against *Candida albicans*, it is plausible that such interaction contributes to the inhibition of this enzyme.

Molecular docking studies in the literature for similar Zn(II) Schiff base complexes provide a predictive framework that aligns with our experimental findings. Saravanaselvam et al. [12] demonstrated through in silico analysis that these complexes exhibit high binding affinity for DNA, which corroborates our in vitro observations of DNA damage. Likewise, Souza et al. [34] performed molecular docking of Zn(II) complexes bearing N,N,S‐donor ligands against the CYP51 enzyme (sterol 14α‐demethylase) of *C. albicans* and *C. glabrata*. Their results demonstrated high affinity and orientation within the enzyme's active site, suggesting that the inhibition of ergosterol biosynthesis is a viable pathway for this class of compounds.

Rani et al. [33] evaluated in silico the ADMET profile of a Schiff base complex with zinc (II), with the aim of investigating its pharmaceutical efficacy in biological systems. The authors observed that the inorganic complex presented favorable parameters for oral administration, indicating potential for the development of new antifungal agents. In this context, considering the structural similarities and the common metal center, it is plausible that the SBZ compound exhibits similar pharmacokinetic behavior, reinforcing its potential as a promising candidate for future antifungal formulations.

Furthermore, Azadi et al. [24] demonstrated the potent antimicrobial activity of a nanoparticle containing a Schiff base complexed with zinc (II) as well as its promising in vitro therapeutic potential in inhibiting the formation of *Candida albicans* biofilms. Based on these findings, future studies should investigate the antifungal potential and safety of SBZ application in vivo for the treatment of candidiasis, especially those caused by FLC‐resistant *Candida* spp. strains, as well as cryptococcosis.

## 3 Conclusion

The advance of resistance to currently available antifungal agents reinforces the need to develop new therapeutic strategies. In this context, metal drugs, especially zinc (II) complexes with Schiff bases, stand out for their potential to act through multiple mechanisms and for their greater biological efficacy in comparison with isolated metal ligands or ions. The Schiff base zinc (II) coordination evaluated in this study demonstrated relevant antifungal activity, consolidating the SBZ complex as a promising antifungal candidate. The results showed that SBZ exhibited significant in vitro antifungal activity against fluconazole‐sensitive and fluconazole‐resistant *Candida* spp. species, as well as against *Cryptococcus neoformans* strains. In addition, SBZ demonstrated additive interactions when combined with fluconazole and itraconazole. Mechanistic studies have indicated that the complex can induce mitochondrial depolarization, increased production of reactive oxygen species, and DNA damage, culminating in phosphatidylserine externalization, reduced cell viability, and induction of apoptosis in *Candida albicans* and *Cryptococcus neoformans* fungal cells. Given these findings, future in vivo studies are essential to confirm the therapeutic efficacy and safety of SBZ, aiming at its potential clinical application in the treatment of candidiasis and cryptococcosis.

## 4 Experimental Section

### 4.1 Zn (II) Complex With Vanillin‐Derived Schiff Base (SBZ) and Analyzed Antifungals

Both the syntheses and characterizations of the Schiff base, obtained from tris(hydroxymethyl)aminomethane and vanillin molecules, and the Zn (II) complex with the Schiff base (SBZ) were performed as described by de Oliveira Pinheiro et al. [19].

### 4.2 Synthesis of Schiff Base Ligand, VTRIS

The Schiff base VTRIS (2‐(((4‐hidroxi‐3‐metoxifenil)metileno) amino)‐2‐(hidroximetil)‐1,3‐propanodiol) was synthesized was brought to low temperature and after 24 h the precipitate was obtained. The compound was obtained and stored in a desiccator under vacuum (yield: 67%).

### 4.3 Synthesis of Zn (II) Acetate Complex With Schiff Base, [Zn(VTRIS)_2_](CH_3_COO)_2_

The Zn complex Schiff base VTRIS was synthesized through complexation reaction. 0.069 g of the ligand, Schiff base VTRIS, were dissolved in 10 mL of distilled water and the Zn (CH_3_COO)_2_ salt (Dynamics) (CAS 5970‐45‐6), 0.030 g, dissolved in 10 mL of distilled water, in a 2:1 ratio of Schiff base and Zn (CH_3_COO)_2_ salt, respectively. The solutions were added to a 50 mL round‐bottom flask under stirring at room temperature. After 20 min of reaction, 3 drops of triethylamine (Synth) (CAS 121‐44‐8) were added to the solution. The reagent solution was stirred for 3 h. After this period and completion of the reaction, the solution was left at low temperature for 48 h until it completely precipitated, filtered, and stored in a desiccator under vacuum, obtaining a light yellow colored salt (yield: 43%).

### 4.4 Spectroscopic Measurements

The absorption spectra in the UV–vis region of the Schiff base VTRIS and the complex [Zn(VTRIS)_2_](CH_3_COO)_2_ in the range 200–800 nm, in water and dimethylsulfoxide (DMSO) (Dynamics) (CAS 67‐68‐5) solvent, respectively, were performed in a Shimadzu 1800 UV–vis spectrophotometer. The measurements of the solutions were made in a quartz cuvette with an optical path of 1 cm in solutions with a concentration of 1 × 10^−3^ mol/L. The molar absorptivity of the ligand and the complex was calculated by Lambert–Beer's law [32].

Spectra in the infrared region were obtained using a Nicolet iS5 spectrophotometer from Thermo Scientific. The samples were prepared as KBr pallets (Synth) (CAS 3 February 7758) in the ratio 1:20 (m/m) (sample: KBr), and the spectra were recorded in the range of 4000 to 400 cm^−1^ using 32 scans and resolution of 4 cm^−1^.

NMR experiments were performed on an Agilent 600 MHz spectrometer equipped with 5 mm inverse detection (H‐F/15N‐31P) One probe with actively protected z‐gradient. First, the sample was optimized with respect to the deuterated solvent (Dimethylsulfoxide–Sigma–Aldrich 99.8%). Samples were suspended in 600 µL of deuterated solvent. All samples were prepared in triplicates. All measurements were carried out at ambient temperature and pressure. The spectra were referenced to resonances of the non‐deuterated residual solvent at δH 2.50 and δC 39.40 for the experiment in dimethylsulfoxide.

In antifungal tests, Schiff base and the SBZ complex were diluted in sterile distilled water and 100% dimethyl sulfoxide (DMSO) (final concentration less than 2.5%), respectively. Fluconazole and itraconazole obtained from Sigma–Aldrich (USA) were used as antifungals, which were diluted in sterile distilled water and 100% DMSO (final concentration less than 2.5%), respectively [19, 35].

### 4.5 Microorganisms Used

Eight strains of *Candida* spp. were used, namely: *Candida parapsilosis* ATCC 22019, *Candida krusei* ATCC 6258, *Candida auris* 01256P derived from CDC B11903, and five clinical strains resistant to fluconazole, including two *Candida albicans*, one *Candida parapsilosis*, one *Candida tropicalis*, and one *Candida glabrata*. Seven clinical strains of *Cryptococcus neoformans* were also used. All strains belong to the Laboratory for Bioprospecting of Antimicrobial Molecules (LABIMAN) at Federal University of Ceará (UFC). The strains were seeded in Sabouraud dextrose agar (HiMedia, Mumbai, India) for 24 h at 35°C for *Candida* spp. and 72 h at 35°C for *Cryptococcus neoformans* [19].

### 4.6 In Vitro Antifungal Susceptibility Testing

The broth microdilution assay in 96‐well polystyrene plates was performed according to protocol M27‐A3 of the Clinical and Laboratory Standards Institute [36]. Roswell Park Memorial Institute (RPMI 1640) culture medium (pH 7.0 ± 0.1) buffered with 0.165 M morpholinopropanesulfonic acid (MOPS) (Sigma, USA) was used. The Schiff base and SBZ complex were tested at concentrations ranging from 1024 to 2 µg/mL. Fluconazole was tested at concentrations ranging from 64 to 0.125 µg/mL, and itraconazole at concentrations ranging from 16 to 0.03125 µg/mL. Inocula were prepared on a McFarland 0.5 scale (0.5 to 2.5 × 10^3^ CFU/mL). After serial dilution of the drugs and addition of the inocula, the plates were incubated for 24 h at 35°C (*Candida* spp.) and for 72 h at 35°C (*Cryptococcus neoformans*). The minimum inhibitory concentration (MIC) was determined as the lowest concentration capable of inhibiting 50% of fungal growth (MIC 50%). For *Candida* spp. strains, those with an MIC ≥ 8 µg/mL were considered resistant to fluconazole [37].

### 4.7 Checkerboard Test

After determining the MIC values for each drug, we performed a checkerboard assay of the SBZ complex with the antifungals fluconazole and itraconazole [38]. The fractional inhibitory concentration index (FICI) was determined by the equation:

$$\begin{array}{ccc} FIC^{A}+FIC^{B} & = & \left(MI{C_{A}}^{COMBINED}/MI{C_{A}}^{ISOLATED}\right)\\ & & +\left(MI{C_{B}}^{COMBINED}/MI{C_{B}}^{ISOLATED}\right) \end{array}$$

whereas MIC_A_^ISOLATED^ and MIC_B_^ISOLATED^ represent the inhibitory concentrations of the isolated drugs, and MIC_A_^COMBINED^ and MIC_B_^COMBINED^ represent the concentrations of the combined drugs.

The interaction results were classified as synergistic (ICIF ≤ 0.5), additive (0.5 < ICIF ≤ 1.0), indifferent (1 < ICIF ≤ 4.0), or antagonistic (ICIF > 4.0) [39].

### 4.8 Flow Cytometry

The strains *Candida albicans* 1 and *Cryptococcus neoformans* 1 were chosen as representatives of each species. Initially, the strains were plated on Sabouraud dextrose agar and incubated for 24 h at 35°C (*Candida albicans*) and for 72 h at 35°C (*Cryptococcus neoformans*). They were then suspended in yeast nitrogen dextrose (YND) and incubated under the same conditions described above. Subsequently, the cells were centrifuged (3 000 g for 5 min), washed with 0.85% saline solution, and the inocula were prepared in RPMI 1640 at a concentration of 10^6^ cells/mL [35, 40].

The treatments performed were SBZ complex at the MIC for each species and fluconazole at the MIC for each species, in addition to the negative control (RPMI 1640 medium only), positive control (strains in RPMI 1640 medium without treatment), and amphotericin B (Ampho B; 4 µg/mL) as a death control for both species. After 24 h of treatment, cell viability, mitochondrial transmembrane potential, reactive oxygen species (ROS) production, and cell labeling with Annexin V assays were performed [35, 40].

#### 4.8.1 Cell Viability

The propidium iodide (PI; 2 µg/mL) exclusion assay was used to assess cell viability after treatment, as described by da Silva et al. [41]. Fluorescence was analyzed using a FACSCalibur flow cytometer (Becton Dickinson, San Jose, CA, USA).

#### 4.8.2 Mitochondrial Transmembrane Potential (Δψm)

To evaluate possible mitochondrial dysfunction caused by the treatments, rhodamine 123 (1 µg/mL) was used, as described by da Silva et al. [41] and Neto et al. [42]. Fluorescence was also analyzed with the FACSCalibur device.

#### 4.8.3 Production of Reactive Oxygen Species (ROS)

ROS production was determined after incubating cells with 20 µmol/L CM‐H_2_DCFDA [5‐(e‐6)‐chloromethyl‐2',7'‐dichlorodihydrofluorescein diacetate acetyl ester], as described by da Silva et al. [41] and Neto et al. [42]. Fluorescence was analyzed by the FACSCalibur.

#### 4.8.4 Cells Stained With Annexin V

To evaluate Annexin V‐positive cells, the FITC‐Annexin V apoptosis detection kit (Guava Nexin Kit, Guava Technologies) was used, as described by da Silva et al. [41] and Neto et al. [42]. Fluorescence was analyzed by the FACSCalibur.

### 4.9 Alkaline Comet Assay

After 24 h with the same treatments used in the flow cytometry assay, the alkaline comet assay was used to evaluate the damage caused to the DNA of *Candida albicans* and *Cryptococcus neoformans*, as described by da Silva et al. [43] and da Silva et al. [35]. The damage index was considered to range from 0 (completely undamaged: 100 cells x 0) to 400 (maximum damage: 100 cells x 4) [41, 42].

### 4.10 Statistical Analysis

All tests were performed in independent triplicates. MIC values were obtained by the arithmetic mean of the triplicate results. The results of the flow cytometry and alkaline comet assays were obtained by one‐way analysis of variance (ANOVA) followed by the Newman–Keuls test using the software GraphPad Prism 5.0, considering *p* < 0.05.

## Author Contributions

**Lívia Gurgel do Amaral Valente Sá**: conceptualization, formal analysis, writing – review and editing. **Cecília Rocha da Silva**: formal analysis, writing – review and editing. **João Batista de Andrade Neto**: formal analysis, writing – review and editing. **Daniel Sampaio Rodrigues**: formal analysis, investigation, methodology, writing – review and editing. **Lara Elloyse Almeida Moreira Gomes**: formal analysis, investigation, methodology, writing – review and editing. **Vitória Pessoa de Farias Cabral**: formal analysis, investigation, methodology, writing – original draft, writing – review and editing. **Wildson Max Barbosa da Silva**: formal analysis, investigation, methodology, writing – review and editing. **Solange de Oliveira Pinheiro**: conceptualization, formal analysis, investigation, methodology, writing – review and editing. **Jordana Lima Braga**: formal analysis, methodology. **Bruno Coêlho Calvancanti**: formal analysis, methodology, writing – review. **Manoel Odorico de Moraes**: formal analysis, writing – review. **Thais Lima Ferreira**: formal analysis, methodology, writing – original draft, writing – review and editing. **Elaine Aires de Lima**: formal analysis, methodology. **Ana Carolina Medeiros de Oliveira**: formal analysis, methodology. **Hélio Vitoriano Nobre Júnior**: conceptualization, project administration, supervision.

## Funding

No funding was received for conducting this study.

## Conflicts of Interest

The authors declare no conflicts of interest.

## Data Availability

All generated data are available in the article.
